# Tisagenlecleucel in Children and Young Adults: Reverse Translational Research by Using Real-World Safety Data

**DOI:** 10.3390/ph13090258

**Published:** 2020-09-21

**Authors:** Concetta Rafaniello, Carmen Ferrajolo, Mario Gaio, Alessia Zinzi, Cristina Scavone, Maria Giuseppa Sullo, Francesco Rossi, Liberato Berrino, Annalisa Capuano

**Affiliations:** 1 Section of Pharmacology “L. Donatelli”, Department of Experimental Medicine, Campania Regional Centre for Pharmacovigilance and Pharmacoepidemiology, University of Campania “Luigi Vanvitelli”, Via Costantinopoli 16, 80138 Naples, Italy; concetta.rafaniello@unicampania.it (C.R.); carmen.ferrajolo@unicampania.it (C.F.); alessia.zinzi@unicampania.it (A.Z.); cristina.scavone@unicampania.it (C.S.); pina.sullo@libero.it (M.G.S.); francesco.rossi@unicampania.it (F.R.); annalisa.capuano@unicampania.it (A.C.); 2 Section of Pharmacology “L. Donatelli”, Department of Experimental Medicine, University of Campania “Luigi Vanvitelli”, Via Costantinopoli 16, 80138 Naples, Italy; liberato.berrino@unicampania.it

**Keywords:** leukaemia, paediatrics, translational medical research, CAR-T cell therapy, safety monitoring

## Abstract

Tisagenlecleucel has revolutionized the pharmacological approach of relapsed or refractory B-cell acute lymphoblastic leukaemialeukaemia in paediatrics. The safety profile of tisagenlecleucel still needs to be better defined. The aim of this study was a post-marketing evaluation of the safety of tisagenlecleucel through the analysis of the Eudravigilance database with focus on the paediatric population. From 2017 to 2020, one third of Individual Case Safety Reports referring to tisagenlecleucel (117/364) have been collected in paediatrics, on average nine year-old boys. Overall, 92% of the638 adverse events were serious and caused or prolonged hospitalisation. A total of 55 adverse events presented a fatal outcome, mainly due to progression of malignant neoplasm (*N* = 10; 18.2%), recurrence of acute lymphocytic leukaemia (*N* = 6; 10.9%) or occurrence of acute lymphocytic leukaemia (*N* = 5; 9.1%). Cytokine release syndrome was commonly reported after tisagenlecleucel infusion (54/638), followed by pyrexia (45/638) and hypotension (27/638). Only 18/638 events referred to neurotoxicity, none of them resulted in death. More than one third of cases (41/117) were suggestive of therapeutic failure. This first post-marketing analysis confirms pre-approval evidence of the safety profile of tisagenlecleucel in paediatrics. Since only a few years of marketing is available, further followed-up studies need to be performed to investigate longer-term safety of tisagenlecleucel.

## 1. Introduction

The development of second-generation CAR-T and the choice of CD19 as a tumour antigen significantly increased CAR-T activity in preclinical studies that were translated into unprecedented clinical results in B-cell acute lymphoblastic leukaemia (B-ALL) and non-Hodgkin lymphoma (NHL) [1,2,3,4,5,6,7,8,9]. CD19 is an attractive target for B-cell neoplasms because expression of CD19 is restricted to normal and malignant B cells and B-cell precursors [10]. Tisagenlecleucel was the first gene therapy to receive approval from the FDA. In August 2017, it was initially approved for the treatment of relapsed or refractory (r/r) paediatric and young-adult B-cell acute lymphoblastic leukaemia (B-ALL), and then, in May 2018, for the treatment of adult patients with r/r diffuse large B-cell lymphoma (DLBCL) [11,12]. Tisagenlecleucel was lately authorized for the treatment of r/r B-ALL and DLBCL also in the European Union (EU) [13]. The first-in-human study of tisagenlecleucel, hereafter referred to as anti-CD19 CAR-T cell, was performed in 2010 only in adults with r/r CD19+ B-cell leukaemia and lymphomas (CART19 phase I trial (NCT01029366)), while the first results of its use in two children were published in 2013, showing a complete remission of the B-ALL [8,14]. Later, a pilot clinical trial on 30 children and adults with r/r ALL treated with anti-CD19 CAR-T cells showed a complete response in 90% of the study population, a 6-month event-free survival of 67% and Overall Survival of 78% [15]. The feasibility and the efficacy of anti-CD19 CAR-T cells was demonstrated through the study ENSIGN, a phase II multicenter trial (NCT02228096) enrolling paediatric and young adults with r/r B-ALL, showing a high overall remission rate (ORR) with durable remissions [16]. However, ELIANA study (NCT02435849), led to approval of tisagenlecleucel in r/r B-ALL patients up to 25 years of age [17]. The study enrolled 75 paediatric and adult subjects with r/r B-ALL. Nonhematologic adverse events observed among study population were mainly cytokine release syndrome (CRS), pyrexia, decreased appetite, febrile neutropenia, and headache. Moreover, in 89% of the study population, adverse events included cytopaenias, infections–pathogen unspecified, viral infectious disorders, neurologic events, and tumour lysis syndrome. Neurotoxicity was observed in 40% of the enrolled patients. Moreover, severe infections, prolonged neutropenia, congestive heart failure, prolonged B-cell aplasia, bleeding, and cerebral haemorrhage were also observed. Even though tisagenlecleucel has revolutionized the pharmacological approach in r/r B-ALL children, its marketing authorisation took in to account findings of small-sized and short-term follow-up trials. Moreover, there is a need to further characterise also identified complications which include prolonged cytopaenias, hypogammaglobulinaemia, risk of secondary malignancies and both neurological and autoimmune disease.

In this scenario, monitoring drug adverse events in post-marketing phase in order to confirm/refuse clinical pre-approval data in the real-world setting is essential [18,19]. Moreover, close post-marketing surveillance becomes mandatory for tisagenlecleucel, as it is reported in the risk management plan (RMP) [20]. Waiting for further studies and ad-interim results from CCTL019B2401 non-interventional PASS, the aim of this study was a post-marketing evaluation of the safety of tisagenlecleucel with a focus on the paediatric population, through the analysis of the EudraVigilance database.

## 2. Results

A total of 364 individual case safety reports (ICSRs) collected from 2017 to 2020 in the EV database were associated to tisagenlecleucel, 117 out of them (32.7%) referred to paediatric patients. After exclusion of 14 ICSRs with gender/age missing information, most of the ICSRs (*N* = 57; 55.3%) were reported in male patients, and the mean age was 9 (±4.6) years. Moreover, the majority of the ICSRs (*N* = 90; 76.9%) came from a Non-European economic area (N-EEA) and were issued by a health care professional (HCP) (92.3%) (Table 1).

Around 80% of ICSRs included more than one AE (median 3.0; standard deviation ±7.2), thus the overall 117 paediatric ICSRs accounted for a total of 638 AEs, the majority of them (91.9%) were classified as serious. Caused/Prolonged Hospitalisation (36.4%) was the seriousness criterium more frequently selected, followed by other medically important condition (32.7%), results in death (12.7%) and disabling (5.7%). Moreover, death has been reported in 24 out of 117 ICSRs (20.5%). The adverse event more frequently reported as cause of death was the progression of disease (i.e., malignant neoplasm progression), acute lymphocytic leukaemia recurrence or acute lymphocytic leukaemia.

Looking at indications of use as reported in the analysed ICSRs, tisagenlecleucel has been used mostly for the treatment of acute lymphoblastic leukaemia in 92 cases (80.7%), 29 of them for the acute lymphoblastic leukaemia recurrence (25.4%) (Table 2), in line with the approved indications.

**Figure 1:**
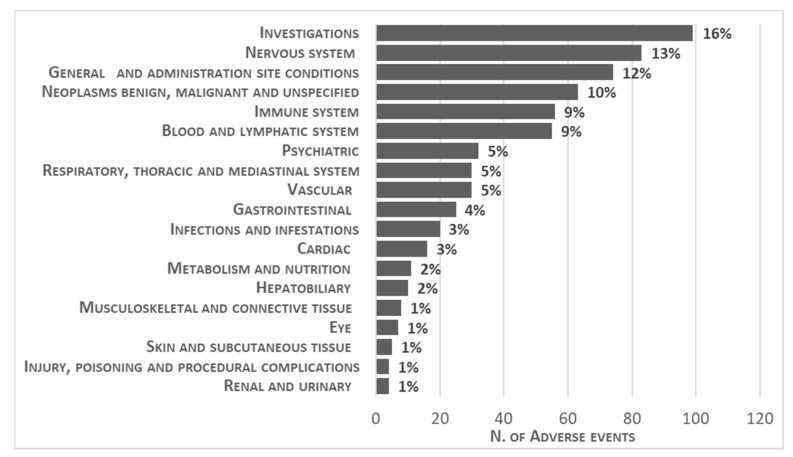
Distribution of System Organ Class related to each adverse event (*n* = 638) reported in overall tisagenlecleucel paediatric Individual Case Safety Reports. Product issue, surgical procedures and disorders involving ear and endocrine systems have been not included in the figure because they accounted for less than 1% of the ICSRs.

The distribution of AEs has been categorized by System Organ Class (SOC) in Figure 1.

In particular, adverse events belonging to the following SOCs, namely Investigations (15.5%), General disorders and administration site conditions (11.6%), Nervous system disorders (11.1%), Blood and lymphatic system disorders (10.5%), Neoplasms benign, malignant (9.9%) and Immune system disorders (8.8%) were reported in more than 60% of the ICSRs. As described in Table 3, within each SOC category, except Investigation, a specific event was more commonly reported than others. For instance, “pyrexia” was the most frequently reported adverse event within general disorders and administration site conditions, “neurotoxicity” within nervous system disorders, as well as “CRS” within Immune system disorders, as expected. Then, neutropenia, anaemia, coagulopathy, B-cell aplasia, thrombocytopenia and febrile neutropenia accounted for more than half of events among blood and lymphatic system disorders. Finally, acute lymphocytic leukaemia recurrence, malignant neoplasm progression and acute lymphocytic leukaemia were the most frequent adverse events among Neoplasms benign, malignant SOC.

As subgroup analysis, 41 out of 117 ICSRs (35%) have been suggested as potential cases of therapeutic failure, accounting for 53 AEs suggestive for therapeutic failure, according to our categorisation. Acute lymphocytic leukaemia recurrence (49.1%), malignant neoplasm progression (34.5%) and therapy non-responder (9.1%) were the most reported events. Conversely, B-cell aplasia, selected as a potential case of therapeutic response, was reported as an AE in eight out of 117 ICSRs (data not shown). Finally, anti-infectives for systemic use, drugs acting on the nervous system and those acting on the gastrointestinal tract and on metabolism were the most frequently reported medicines as concomitant therapy (Table 4).

## 3. Discussion

In this study, we investigated spontaneous reports of adverse events related to tisagenlecleucel in a paediatric population. To our knowledge this is the first study that analyses the safety data coming from the “real world”. One third of ADRs referred to tisagenlecleucel have been collected over a four-year post-marketing period in children, predominantly nine-year-old boys. The majority of adverse events were serious and caused or prolonged hospitalisation, according to the severity of existing illness.

Tisagenlecleucel, the first CAR-T cell approved for the treatment of relapsed or refractory (r/r) B-ALL in children and young adults (later also for the treatment of r/r diffuse large B-cell lymphoma in adults) has successfully filled a therapeutic gap for this life-threatening disease which mostly affects children and adolescents [21]. However, as often occurs, therapies that lead to a revolution in the history of the management of rare and life-threatening diseases, are generally associated with a new safety concern.

In this regard, as additional pharmacovigilance activities in RMP, the tisagenlecleucel’s Marketing Authorization Holder has established a protocol for a non-interventional post-authorization safety study with secondary use of data from two registries [20]. The ongoing study, applied in the EU-PAS register (ni-PASS CCTL019B2401), is being conducted by the “European Society for Blood and Marrow Transplantation” (EBMT) and “Centre for International Blood and Marrow Transplant Research” (CIBMTR) with the aim to evaluate in a real-world setting the long-term safety of patients with B lymphocyte malignancies treated with tisagenlecleucel. In this scenario, a rapid and careful analysis of the spontaneous ADR reporting database may provide a prompt overview of first safety data after a few years of marketing of tisagenlecleucel, while waiting for results from the aforementioned PAS study.

The toxicity mostly associated to tisagenlecleucel is the CRS resulting from release of cytokines and chemokines from T cell activation when their CARs engage the CD19 antigen on the malignant B-cells. In particular, IL-6, soluble IL-6 receptor, soluble IL-2 receptor alpha, interferon gamma (IFN-γ), and granulocyte-macrophage colony-stimulating factor (GMCSF) increase in patients with severe CRS [22]. Clinical signs of CRS are fever, hypotension, hypoxia and tachycardia, but it may also occur as coagulopathic, cardiac, hepatic and renal disorders [23]. Consistent with available clinical evidence, our results show that CRS is the most common toxicity from tisagenlecleucel infusion. Specifically, although CRS has been reported as a specific immunological event in less than half of paediatric ICSRs, the other signs related to CRS have been described as adverse events in many more ICSRs. Indeed, pyrexia, which is usually defined as the first presenting sign of CRS, was the most commonly reported AE belonging to the SOC General disorders and administration site conditions. Moreover, a serum ferritin increase, C-reactive protein increase, and fibrin D dimer increase have been the most frequently reported AEs among the “investigations” SOC, in line with the blood tests of systematic inflammation response in the CRS. In only one case did the CRS lead to death; however, other serious AEs which have likely contributed to the fatal outcome, such as cardiac arrest, mental status changes, neurotoxicity, oxygen saturation decrease, respiratory failure and sinus tachycardia, have been reported in the same case. All these events have been well-described together with CRS; specifically, CRS severity stage, i.e., CRS leading to potentially life-threatening vasodilatory shock, capillary leak, hypoxia up to organ dysfunction, has been observed even in the paediatric population [24]. Nevertheless, only one death due to CRS has been reported after tisagenlecleucel infusion in the ELIANA clinical trial [17].

Neurotoxicity represents another common toxicity related to tisagenlecleuel, better defined as Immune effector cell-associated neurologic syndrome (ICANS) as recommended by the consensus of the American Society for Transplantation and Cellular Therapy (ASTCT; formerly American Society for Blood and Marrow Transplantation, ASBMT) [25]. As documented from a clinical trial in patients with leukaemia, the occurrence of ICANS ranges between 40% to 62% of patients treated with CAR-T cells [22]. Overall, neurologic adverse reactions reported with tisagenlecleucel are headache, encephalopathy, delirium, anxiety and sleep disorders. Neurotoxicity is usually reversible, however cerebral oedema and/or death have been documented [26]. Although the pathophysiology of neurotoxicity is not yet well understood, it has been suggested that neurologic adverse events may result from severe CRS. Specifically, capillary leak and endothelial activation caused by CRS lead to an increased permeability of the blood brain barrier, allowing the influx of inflammation mediators from the blood to the central nervous system [22,27]. This proposed mechanism has been then confirmed through the identification of proteins and elevated levels of cytokines in the cerebrospinal fluid (CSF) of patients with neurotoxicity. However, ICANS can occur concurrently with CRS, following its resolution, or without associated CRS, likely suggesting an independent mechanism [22,27]. Referring to the paediatric population, as reported in the ELIANA trial, neurotoxicity was observed in 40% of the study population [17]. In this regard, our analysis highlighted neurotoxicity as a specific adverse event in 18/117 ICSRs, although neurological signs have been reported in other several cases. Moreover, neurotoxicity was reported together with CRS in 13/18 cases, confirming results from the above-mentioned evidence [22,27]. Additionally, in line with pre-marketing data, no fatal outcome has been reported for neurotoxicity, alone or in combination with CRS [17,27].

Although a remarkable drug response (1-year overall survival = 76%, overall remission rate = 81% and complete remission = 60%) as shown from the ELIANA trial, relapse and/or the lack of response remain a concern [17]. Actually, loss of CAR-T cell persistence, or relapse of CD19-negative disease are described as the main mechanisms involved in treatment resistance [28]. Consistently, our results highlighted that more than 40% of adverse events referring to recurrence of lymphocytic leukaemia or progression of malignant neoplasm are suggestive of therapeutic failure, most of them even resulted in death. Indeed, our sub-analysis on fatal outcome confirmed that 21% of the 24 cases of death is likely due to therapeutic failure. Although these real-world safety data are not comparable with the ones from clinical trials, our results are quite in line, even if slightly higher, with the Eliana trial results in which 15% of treated patients (11/75) died, and, even more interestingly, seven of them died from disease progression, similarly to our observation. Obviously, our data need to be confirmed by more prolonged follow-up studies to better define the mechanisms underlying the lack of response and the relapses as well as to identify any predictive factors or specific efficacy biomarkers in the paediatric population.

Last but not least, B-cell aplasia resulting in hypogammaglobulinemia is one of the most common late events induced by CAR-T cell therapy. In particular, it is an expected result of successful CD19-specific CAR T-cell treatment due to the selectivity of tisagenlecleucel toward CD19 antigene-expressing cells irrespective of their malignancy. The hypogammaglobulinemia is due to the destruction of normal beta-cell expressing CD-19 and it represents an efficacy therapy persistence marker in treated patients. In other words, B-cell aplasia can be considered as a useful pharmacodynamics biomarker of CAR-T cell therapy functional persistence instead of an adverse event; in fact, its loss has been associated with an increased risk of relapse [23]. However, if B-cell aplasia seems to be well tolerated or otherwise overcome with immunoglobulin replacement given the lack of humoral adaptive immunity, the long-term effects of prolonged B-cell aplasia and resulting hypogammaglobulinemia have not yet well defined. We found only a few cases (8/117) reporting B-cell aplasia, not exactly matching the frequency described in available scientific evidence [17,29]. It may depend on the fact that B-cell aplasia is not properly considered as an adverse event, but rather as an expected one unless it became prolonged, leading to hypogammaglobulinemia; in addition, our findings could be due the appropriate use of immunoglobulin replace as supportive care for children with B-cell aplasia [30,31]. Moreover, recently, B-cell aplasia has been included as uncommon adverse drug reaction (frequency ranked ≥1/1000 to <1/100) in the last updated Summary of the Product Characteristics (SPC) of tisagenlecleucel (updated on 1 April 2020), likely suggesting a low frequency of occurrence or a challenging identification as adverse event. However, the “other side” of this type of expectable toxicity, referred to “on-target (targeting the CD19 antigen), off-tumour toxicity” associated to CAR-T cell is that as long as hypogammaglobulinemia persists, risk of infections increases as well [32]. In this respect, any case referred to hypogammaglobulinemia as an AE has been observed in our database.

Concerning the proper use of tisagenlecleucel, we would point out that this drug has been used for approved indications, except in four cases where B-cell lymphoma recurrence was the reported clinical indication. As reported in the SPC, tisagenlecleucel is indicated in adult patients with relapsed or refractory diffuse large B-cell lymphoma (DLBCL) but not in the paediatric population.

Noteworthy, according to the RMP, newly diagnosed malignancies are one of the major concerns associated to CD19 CAR-T cell use. Nevertheless, we found only one case referring to “Second primary malignancy”. In line with the long latency of disease, longer-term safety data need to be collected and investigated.

This study has several strengths. Firstly, to the best of our knowledge, this is the first report of post-marketing surveillance of the safety profile of tisagenlecleucel, by using data from ADR spontaneous reports. This system reflects both real-life events and real-life drug use, including drug use patterns that cannot be studied in clinical trials for ethical reasons. Moreover, Eudravigilance is one of the largest databases of spontaneous reports, covering heterogeneous information from different countries and populations. Although data from spontaneous reporting adverse drug reactions could be useful to promptly identify (new) safety signals, several limitations need to be considered while interpreting this type of result. Firstly, underreporting is highly probably in a spontaneous report system, and the reported adverse events can represent only the tip of the iceberg, mainly in paediatric patients [33,34]. The number of reports, indeed, can vary due to several reasons, such as a different attitude to the reporting activities. This is particularly true for healthcare professionals working in special departments with more vulnerable patients, that is during their daily clinical practice they could pay more attention to unexpected and serious adverse events, rather than to serious but well-established ones on the basis of their clinical experience [35]. Moreover, with this type of data, no information on exposed population is available, so that it is not possible to have a denominator for comparison. Another challenge of this type of study is that the MedDRA terms do not always match exactly the adverse reactions as defined in the SmPC and literature. In order to avoid misclassification of events, we categorized signs and symptoms based on clinical expertise. Finally, since restricted data elements were provided from the spontaneous reporting systems database with an on-line access tool, additional information useful to well define the event and the outcome, such as severity of underlying illness, causality assessment, as well as ADR narrative or follow-up, were unavailable [36].

## 4. Materials and Methods

Individual case safety reports (ICSRs) in the EudraVigilance (EV) database spontaneously reported by a healthcare professional (HCP) or non-HCP to an EU national competent authority (NCA) or marketing application holder (MAH) from 1 January 2017 through 25 March 2020 have been retrieved. EV is a centralized European database, maintained by the European Medicines Agency (EMA), containing all spontaneous ICSRs of suspected adverse events (AE) related to a drug or a vaccine authorized in the European Economic Area (EEA). Aggregate data using the public access online tools adverse drug reaction (ADR)-website are available as line listing and ICSR from (http://www.adrreports.eu/en/search.html). Specifically, the ADR-website provides the possibility to retrieve data for the individual cases based on some data elements relative to patient characteristics (age and gender), reactions/events (event, duration, outcome and seriousness), drugs (role, products or substance, duration of use, dose, units in interval, action taken, indication, pharmaceutical form and route of administration) and general information such as EV Local Report Number, primary source country and reporter’s qualification [36]. For this analysis all ICSRs associated to tisagenlecleucel and referring to the paediatric population aged 0–17 years have been selected.

In order to perform a descriptive analysis as complete as possible, we obtained the following information reported in both line listing and the ICSR form and related to patient, such as gender and age, type of reporter (HPC or non-HPC), area of report origin (EEA and Non-EEA), reported AEs, suspected and concomitant (including interacting) drugs. Since each ICSR can contain more than one AE, the total number of AEs will be higher than the overall ICSRs.

Reported AEs are coded according to the Medical Dictionary for Regulatory Activities (MedDRA) and analysed based on preferred terms (PT) and corresponding System Organ Class (SOC).

Seriousness of reported AEs has been also investigated. Among serious AEs “resulted in death”, cases with fatal outcome were investigated as sub-analysis.

Moreover, all ICSRs including at least one AE potentially suggestive for drug response or failure were considered. Accordingly, we categorised a specific adverse event as follows: “B-cell aplasia” as drug response, and “Acute lymphocytic leukaemia recurrent”, “Acute lymphocytic leukaemia refractory”, “B-cell lymphoma recurrent”, “Disease progression”, “Malignant neoplasm progression” or “Neoplasm progression” as drug failure, either if reported in combination with “therapy non-responder”, “treatment failure” or “drug ineffective” or not.

Finally, all medicines reported as concurrently administered with tisagenlecleucel have been described.

## 5. Conclusions

This is the first report of post-marketing surveillance of safety profile of tisagenlecleucel, by using data from ADR spontaneous reports. The present analysis provides an overview of spontaneous reports of adverse events occurring in children and adolescents treated with tisagenlecleucel, representing a bridge between pre-marketing data to clinical practice. Results of this four-year period post-marketing surveillance confirm the safety profile as investigated in paediatric pre-marketing clinical trials. Since only a few years of marketing are available, further follow-up studies need to be performed to investigate longer-term safety of this drug.

## Figures and Tables

**Table 1:** Demographic characteristics of Individual Case Safety Reports involving tisagenlecleucel recognized in the Eudravigilance spontaneous reporting system from 2017–2020 in the paediatric population.

Demographic Characteristics of Selected ICSRs	Individual Case Safety Reports *N* = 117 (%)
Gender ^a^	
Male	57 (55.3)
Female	46 (44.7)
Mean Age (SD ^b^)	9 (±4.6)
Report Type	
Spontaneous	117 (100.0)
From studies	-
Primary Source	
Health Care Professional	108 (92.3)
Non- Health Care Professional	9 (7.7)
Country	
European Economic Area	27 (23.1)
Non-European Economic Area	90 (76.9)

^a^ Individual case safety reports with gender information missing (*n* = 14) have been excluded; ^b^ Standard Deviation.

**Table 2:** Distribution of therapeutic indications as reported in tisagenlecleucel paediatric Individual Case Safety Reports.

Indication of Use	ICSRs ^a^ *N* = 114 ^b^ (%)
Acute lymphoblastic leukaemia	63 (55.3)
Acute lymphoblastic leukaemia recurrent	29 (25.4)
Product used for unknown indication	4 (3.5)
B-cell lymphoma recurrent	4 (3.5)
Acute lymphocytic leukaemia recurrent	3 (2.6)
Acute lymphocytic leukaemia refractory	3 (2.6)
B-cell type acute leukaemia	2 (1.8)
Bone marrow disorder	2 (1.8)
Acute lymphocytic leukaemia	1 (0.9)
B-cell lymphocytic lymphoma recurrent	1 (0.9)
Leukaemia recurrent	1 (0.9)
Precursor B-lymphoblastic leukaemia acute	1 (0.9)

^a^ Individual Case Safety Reports; ^b^ Therapeutic indication is not reported in 3 ICSRs.

**Table 3:** Distribution of adverse events (at least 2) belonging to the top 6 System Organ Class (SOC) by preferred terms reported in the ICSRs involving tisagenlecleucel as the suspected drug.

SOC and Preferred Term	Adverse Events *N* (%)
**Investigations**	**99 (100)**
Serum ferritin increased	7 (7.1)
C-reactive protein increased	6 (6.1)
Fibrin D dimer increased	4 (4.0)
Activated partial thromboplastin time prolonged	3 (3.0)
Blood potassium decreased	3 (3.0)
B-lymphocyte count abnormal	3 (3.0)
International normalised ratio increased	3 (3.0)
Blood albumin decreased	2 (2.0)
Blood bilirubin increased	2 (2.0)
Blood calcium decreased	2 (2.0)
Blood fibrinogen increased	2 (2.0)
Blood lactate dehydrogenase increased	2 (2.0)
Blood phosphorus decreased	2 (2.0)
Blood pressure diastolic decreased	2 (2.0)
Haematocrit decreased	2 (2.0)
Haemoglobin decreased	2 (2.0)
Lymphocyte count decreased	2 (2.0)
Platelet count decreased	2 (2.0)
Prothrombin time prolonged	2 (2.0)
Red blood cell count decreased	2 (2.0)
White blood cell count decreased	2 (2.0)
**General disorders and administration site conditions**	**74 (100)**
Pyrexia	45 (60.8)
Death	5 (6.8)
Fatigue	5 (6.8)
Therapy non-responder	3 (4.1)
Disease progression	2 (2.7)
Drug ineffective	2 (2.7)
Multiple organ dysfunction syndrome	2 (2.7)
**Nervous System Disorders**	**71 (100)**
Neurotoxicity	18 (25.4)
Headache	8 (11.3)
Seizure	5 (7.0)
Encephalopathy	4 (5.6)
aphasia	3 (4.2)
Facial paralysis	3 (4.2)
Lethargy	2 (2.8)
**Blood and lymphatic system disorders**	**67 (100)**
Neutropenia	13 (19.4)
Anaemia	12 (17.9)
Coagulopathy	9 (13.4)
B-cell aplasia	8 (11.9)
Thrombocytopenia	8 (11.9)
Febrile neutropenia	7 (10.4)
Bone marrow failure	2 (3.0)
Leukopenia	2 (3.0)
**Neoplasms benign. malignant and unspecified**	**63 (100)**
Acute lymphocytic leukaemia recurrent	27 (42.9)
Malignant neoplasm progression	17 (27.0)
Acute lymphocytic leukaemia	9 (14.3)
Central nervous system leukaemia	2 (3.2)
Minimal residual disease	2 (3.2)
**Immune system disorders**	**56 (100)**
Cytokine release syndrome	54 (96.4)

**Table 4:** Distribution of medicines reported as concomitant with tisagenlecleucel belonging to the top 3 drug classes, grouped by anatomical main group (1st level of ATC) and by therapeutic subgroup (2nd level of ATC).

**Anti-infectives for systemic use (J)**	**62 (23.5)**
Antibacterials for systemic use (J01)	43
Antimycotics for systemic use (J02)	15
Antivirals for systemic use (J03)	2
Antimycobacterials (J04)	1
Immune sera and immunoglobulins (J06)	1
**Nervous system (N)**	**43 (16.3)**
Analgesics (N02)	15
Antiepileptics (N03)	12
Anesthetics (N01)	9
Psycholeptics (N05)	6
Other nervous system drugs (N07)	1
**Alimentary tract and metabolism (A)**	**42 (15.9)**
Antiemetics and antinauseants (A04)	11
Drugs for acid related disorders (A02)	6
Drugs for constipation (A06)	6
Mineral supplements (A12)	6
Drugs for functional gastrointestinal disorders (A03)	3
Bile and liver therapy (A05)	3
Antidiarrheals, intestinal anti-inflammatory/anti-infective agents (A07)	3
Vitamins (A11)	2
Drugs used in diabetes (A10)	1
Other alimentary tract and metabolism products (A16)	1

Drug classes accounting for less than 15% of Individual Case safety Reports have been not included.
